# Combined Analysis of Network Toxicology and Metabolomics Uncovers the Potential Mechanisms Underlying Neodymium Oxide-Induced Pulmonary Fibrosis

**DOI:** 10.3390/toxics14060513

**Published:** 2026-06-12

**Authors:** Kai Wu, Yi Zhang, Xi Chen, Yanhong Dong, Suhua Wang, Yanrong Gao

**Affiliations:** School of Public Health, Baotou Medical College, Inner Mongolia Autonomous Region, Baotou 014040, China; 18590293806@163.com (K.W.); 15614787258@163.com (Y.Z.); chenxi2019hq308@163.com (X.C.); 15104720862@163.com (Y.D.)

**Keywords:** neodymium oxide, pulmonary fibrosis, network toxicology, metabolomics

## Abstract

Rare earth element-related occupational and environmental health risks have received increasing attention, but the molecular mechanisms underlying neodymium oxide (Nd_2_O_3_)-induced pulmonary fibrosis remain unclear. This study aimed to identify potential targets, metabolites, and pathways involved in fibrosis-related lung responses after Nd_2_O_3_ exposure. An integrated network toxicology and metabolomics approach was combined with an in vivo mouse model of Nd_2_O_3_-induced pulmonary toxicity. Histopathological injury, collagen deposition, and fibrosis-related protein expression were evaluated by HE staining, Masson staining, and Western blotting, respectively. Candidate targets were identified from public databases, key pathways were analyzed through network construction, and selected genes were validated by RT-qPCR. Nd_2_O_3_ exposure caused evident lung injury, inflammatory cell infiltration, structural disruption, increased collagen deposition, and elevated fibrosis-related protein expression. A total of 162 overlapping targets related to Nd_2_O_3_ exposure and pulmonary fibrosis were identified. *ESR1*, *PTGS2*, *HSP90AA1*, and *MMP9* emerged as key targets, while cAMP signaling, arachidonic acid metabolism, PI3K-Akt signaling, and efferocytosis-related processes were implicated. Metabolomics showed distinct separation between control and high-exposure groups, with differential metabolites mainly associated with lipid metabolism and inflammation. RT-qPCR further confirmed altered expression of key genes. These findings suggest that Nd_2_O_3_ may promote fibrosis-related lung responses through inflammatory signaling, lipid metabolic disturbance, and profibrotic pathway activation.

## 1. Introduction

Rare earth elements (REEs), including scandium, yttrium, and the fifteen lanthanides, are widely used in advanced technologies because of their unique physicochemical properties [1]. Among them, neodymium (Nd) is particularly important due to its extensive applications in advanced technologies, such as high-performance permanent magnets [2]. Neodymium oxide (Nd_2_O_3_), a critical raw material utilized in the production of electrolytic neodymium, predominantly enters the human body through the respiratory system, typically in the form of fine particulate matter or aerosols. Subsequently, it is transported via the circulatory system to diverse tissues and organs throughout the body. As a result, extended exposure to Nd_2_O_3_, whether through its extraction, production, and processing phases or via its incorporation in pharmaceuticals and industrial products, may lead to significant bioaccumulation and potential toxicity [3]. Our own previous research, along with epidemiological and animal studies, has provided evidence that exposure to Nd_2_O_3_ dust is associated with the development of pneumonia and pulmonary fibrosis [4,5].

Pulmonary fibrosis encompasses a heterogeneous group of fibrotic lung conditions characterized by scarring and remodeling of lung tissue, which may arise from diverse etiological factors, including environmental and occupational exposures, drug toxicity, radiation injury, and autoimmune diseases, or may be idiopathic [6]. The primary pathological features include the proliferation and differentiation of fibroblasts, abnormal accumulation of extracellular matrix (ECM), infiltration of inflammatory cells, and damage to the alveolar structure. Pulmonary fibrosis encompasses several forms, including idiopathic pulmonary fibrosis, allergic pneumonia, pneumoconiosis, sarcoidosis, drug-induced and radiation-induced fibrosis, as well as fibrotic alveolitis associated with collagen vascular diseases [7,8]. In recent years, pulmonary fibrosis has emerged as a significant public health concern due to its markedly increased incidence worldwide. Because the underlying mechanisms of the disease remain unclear, early diagnosis is challenging, and no specific treatment is currently available [9]. The prognosis of pulmonary fibrosis is poor and may even be worse than that of some cancers; in clinical practice, patients often succumb to progressive respiratory failure [10,11].

As modern industry continues to advance, the demand for Nd_2_O_3_ is increasing, raising occupational health concerns [12]. With this growing demand, workers are continually exposed to high concentrations of Nd_2_O_3_ dust during production and processing, thereby increasing their risk of pneumoconiosis [13]. Although the incidence of pneumoconiosis has been significantly reduced through current preventive measures, it continues to affect a substantial number of workers [14]. Studies indicate that Nd_2_O_3_ exerts cytotoxic effects on both cancer and immune cells, and through the lncRNA H19/miR-29a-3p/SNIP1/c-myc regulatory axis, it increases the likelihood of lung injury [15]. Nd_2_O_3_ also influences macrophages by activating the NF-κB signaling pathway, leading to inflammation and potentially contributing to the development of pulmonary fibrosis [16]. However, the molecular mechanisms underlying Nd_2_O_3_-induced pulmonary fibrosis remain poorly understood.

Network toxicology is an interdisciplinary strategy integrating bioinformatics, systems biology, and chemoinformatics [17]. By leveraging multi-source databases and network-based modeling, it predicts and integrates potential targets, signaling pathways, and toxicity-relevant molecular events of a given compound, thereby enabling a systems-level characterization of toxic mechanisms [18]. Compared with traditional toxicology, network toxicology emphasizes system-wide biological responses and the connectivity of toxicity pathways. Nevertheless, its predictive outputs are frequently not comprehensively confirmed by rigorously designed animal experiments. Consequently, network toxicology studies often integrate animal-derived evidence—ranging from in vivo phenotypes, histopathological findings, and biochemical indices to multi-omics data, including transcriptomic and proteomic profiles—to validate and calibrate pivotal toxicological pathways, thereby enhancing the reliability and interpretability of the predictions [19]. Endogenous metabolites are small organic molecules involved in diverse metabolic processes, including organic acids and lipids [20]. Untargeted metabolomics provides an unbiased and powerful approach to profile endogenous metabolites, facilitating the discovery of altered metabolites and potential biomarkers [21]. Because metabolic networks are tightly coupled to redox homeostasis, energy metabolism, and inflammatory–immune regulation, metabolomics is particularly valuable for elucidating complex toxic mechanisms and identifying candidate biomarkers [22]. Given the need to further clarify the cytotoxic effects and underlying mechanisms of Nd_2_O_3_ exposure in pulmonary fibrosis, it is important to apply an integrated network toxicology–metabolomics framework to support mechanistic toxicology research and future risk assessment of Nd_2_O_3_ exposure.

Therefore, the present study aimed to investigate the pulmonary fibrosis-related responses induced by Nd_2_O_3_ exposure and to elucidate the underlying mechanisms from the perspectives of metabolic alterations and molecular regulatory networks. Based on previous evidence, we hypothesized that Nd_2_O_3_-induced lung injury and collagen deposition are closely associated with inflammatory signaling activation, lipid metabolic disturbance, and fibrosis-related molecular regulation. To test this hypothesis, an in vivo mouse model was combined with network toxicology, metabolomics, histopathological analysis, Western blotting, and RT-qPCR validation. The findings showed that Nd_2_O_3_ exposure promoted lung pathological injury, collagen accumulation, and fibrosis-related protein expression, accompanied by alterations in key molecular targets, inflammatory and profibrotic signaling pathways, and lipid metabolism-related metabolites. These results provide an integrated mechanistic perspective on Nd_2_O_3_-induced pulmonary fibrosis from both molecular regulatory and metabolic viewpoints, thereby advancing the understanding of rare earth oxide-associated pulmonary toxicity and supporting the identification of potential biomarkers, molecular targets, and intervention strategies for occupational and environmental health risk assessment.

## 2. Materials and Methods

### 2.1. Nd_2_O_3_ Particles and Preparation for Animal Exposure

Neodymium oxide particles (Nd_2_O_3_) were obtained from Baotou Ruixin Rare Earth Smelter, China. As characterized in our previous study using transmission electron microscopy, dynamic light scattering, and zeta potential analysis, the particles exhibited an irregular polyhedral morphology, with an average particle size of approximately 60 nm, an average hydrodynamic size of 540.20 nm in RPMI-1640 medium, and a zeta potential of 4.26 mV [16]. Before animal exposure, the Nd_2_O_3_ particles were autoclaved at 103.4 kPa (1.05 kg/cm^2^) and 121 °C for 20 min (Esco Enterprise Development, Shanghai, China). The particles were then dried overnight at 60 °C in a forced-air drying oven (Anhe Zhengheng Instrument, Sichuan, China) before use.

### 2.2. Animals and Treatments

Male C57BL/6J mice (6–8 weeks old) were purchased from Sibef (Beijing, China) and acclimated for 1 week at the Experimental Animal Center of Baotou Medical College. All animals were housed under standard conditions with a 12 h light/dark cycle and had free access to food and water. All animal procedures were conducted in accordance with relevant institutional and national guidelines and complied with the Guide for the Care and Use of Laboratory Animals. The study protocol was approved by the Animal Ethics Committee of Baotou Medical College (Approval No. BLYS [2024]006). The 90-day Nd_2_O_3_ exposure experiment was initiated in March 2025, after ethical approval had been obtained. This study was designed to evaluate the effects of Nd_2_O_3_ exposure on lung tissue injury in mice. An Nd_2_O_3_ exposure model was established using a dynamic nose-only inhalation exposure system. During exposure, mice were placed in a custom-made restraint device within the dynamic nose-only inhalation chamber, with their noses and mouths directed toward the rare earth dust generator. Mice were randomly divided into a control group, a 250 mg/m^3^ Nd_2_O_3_ exposure group, a 500 mg/m^3^ Nd_2_O_3_ exposure group, and a 1000 mg/m^3^ Nd_2_O_3_ exposure group, with 12 mice in each group. The exposure concentrations of 250, 500, and 1000 mg/m^3^ were selected to establish a low-, medium-, and high-dose gradient for evaluating the dose-dependent effects of Nd_2_O_3_ exposure. The 1000 mg/m^3^ exposure concentration was selected with reference to a previous inhalation study of Nd_2_O_3_ [5]. Given that this group showed more evident histopathological changes in lung tissue in the present study, it was subsequently chosen for metabolomics analysis. Mice in the exposure groups were exposed to the corresponding concentrations of Nd_2_O_3_ aerosol for 2 h/day for 90 consecutive days. The control group was exposed to filtered air under the same dynamic nose-only inhalation conditions for the same duration, without saline inhalation or Nd_2_O_3_ aerosol exposure. After the final exposure, mice were euthanized and lung tissue samples were collected. The collected lung tissues were used for histopathological, metabolomic, and subsequent molecular biological analyses. Of the 12 mice in each group, 5 mice were randomly selected for histopathological analysis of lung tissue. In addition, 3 mice from the control group and 3 mice from the 1000 mg/m^3^ Nd_2_O_3_ exposure group were randomly selected for untargeted metabolomics analysis. The remaining lung tissue samples were used for subsequent molecular biological analyses. To reduce potential physiological variation associated with the estrous cycle, only male mice were used in this study. Therefore, the findings of this study primarily reflect the response characteristics of male mice to Nd_2_O_3_ exposure and should be interpreted accordingly.

### 2.3. Lung Histopathology

Lung tissues were fixed in 4% paraformaldehyde at room temperature for 24 h, dehydrated through a graded ethanol series, cleared in xylene, embedded in paraffin, and cut into 5 μm thick sections using a rotary microtome (RM2135; Leica, Wetzlar, Germany). For hematoxylin and eosin (H&E) staining, paraffin sections were deparaffinized, rehydrated, stained with hematoxylin, differentiated in acid alcohol, rinsed in running tap water for blueing, counterstained with eosin, dehydrated through a graded ethanol series, cleared in xylene, and mounted with neutral balsam. H&E-stained sections were used to evaluate histopathological alterations in lung tissues. For Masson’s trichrome staining, paraffin sections were deparaffinized, rehydrated, and stained with Weigert’s iron hematoxylin, followed by acid fuchsin staining, phosphomolybdic acid differentiation, and aniline blue counterstaining. After brief differentiation in acetic acid solution, the sections were dehydrated, cleared in xylene, and mounted with neutral balsam. Collagen fibers were stained blue and were used to assess collagen deposition in lung tissues. Images of H&E- and Masson’s trichrome-stained lung sections were captured at ×200 magnification using an Olympus BX43 light microscope equipped with an Olympus UC90 imaging system (Olympus, Tokyo, Japan). Images used for semi-quantitative histomorphometric analysis were acquired under the same magnification and imaging settings. Semi-quantitative histomorphometric analysis of collagen-positive areas was performed using Fiji (ImageJ, version 2.14.0). software. Briefly, 24-bit RGB images of Masson’s trichrome-stained lung sections were imported into ImageJ/Fiji, and the blue collagen signal was separated using the Colour Deconvolution plugin with the Masson Trichrome vector. Threshold segmentation was then applied to generate binary images of collagen-positive areas, and the same threshold settings were applied to all images within the same analysis. The lung parenchymal region was defined as the region of interest, avoiding large bronchi and blood vessels. The collagen-positive area and total lung parenchymal area were measured using the Measure function, and the percentage of collagen-positive area was calculated as follows: collagen-positive area (%) = collagen-positive area/total lung parenchymal area × 100%. For each mouse, the mean value of 25 fields from five sections, with five non-overlapping fields per section, was used for statistical analysis. Histological evaluation and ImageJ/Fiji-based quantification of collagen-positive areas were performed using coded images by investigators blinded to group allocation. Group codes were revealed only after quantitative measurements were completed.

### 2.4. Western Blot Analysis

Proteins were extracted from mouse lung tissues using RIPA lysis buffer containing protease inhibitors, and protein concentrations were determined using a BCA protein assay kit according to the manufacturer’s instructions. Equal amounts of protein from each sample (80 μg) were separated by 10% SDS-PAGE and transferred onto polyvinylidene fluoride membranes. After blocking with 5% non-fat milk for 1 h at room temperature, the membranes were incubated overnight at 4 °C with the following primary antibodies: anti-GAPDH antibody (rabbit, 1:5000, Signalway Antibody/SAB), anti-Collagen I antibody (rabbit, 1:1000, Signalway Antibody/SAB), and anti-α-SMA antibody (rabbit, 1:1000, Signalway Antibody/SAB). After washing three times with TBST, the membranes were incubated with horseradish peroxidase-conjugated goat anti-rabbit IgG secondary antibody (1:20,000, Signalway Antibody/SAB) for 1 h at room temperature. Protein bands were detected using an enhanced chemiluminescence system. Densitometric analysis of the blots was performed using ImageJ software. For each sample, the gray intensity of the Collagen I or α-SMA band was normalized to the gray intensity of the corresponding GAPDH band. The normalized value was then expressed relative to the mean value of the control group, which was set as 1.

### 2.5. Collection of Nd_2_O_3_-Related Targets

To obtain the standard structure and SMILES notation of Nd_2_O_3_, the PubChem database was queried. Using the keyword “Neodymium oxide”, potential targets were collected from the ChEMBL, TargetNet, and SuperPred databases, with the species restricted to Homo sapiens. In addition, the SMILES code of Nd_2_O_3_ was submitted to the STITCH database to identify additional potential targets. All retrieved targets were integrated, and duplicate entries from different databases were removed. To ensure consistency in target annotation, all target names were standardized to official gene symbols using the UniProt database. After integration, de-duplication, and standardization, a comprehensive Nd_2_O_3_-related target dataset was established for subsequent analysis.

### 2.6. Collection of Pulmonary Fibrosis-Related Targets

Pulmonary fibrosis-related targets were collected from the GeneCards, DrugBank, TTD, DisGeNET, and OMIM databases using the keyword “Pulmonary fibrosis”. For databases providing relevance scores, targets with scores above the median value were retained to ensure a relatively high correlation with pulmonary fibrosis. For databases without a unified scoring system, all retrieved targets were included. All targets were then integrated, duplicate entries were removed, and the target names were standardized to official gene symbols using the UniProt database. A Venn diagram was subsequently used to identify the overlapping targets between the Nd_2_O_3_-related target set and the pulmonary fibrosis-related target set, and these common targets were regarded as potential targets of Nd_2_O_3_ affecting pulmonary fibrosis.

### 2.7. Protein Interaction Network Construction and Core Target Screening

The potential target genes associated with Nd_2_O_3_-induced pulmonary fibrosis were submitted to the STRING database, with the species filtered to “Homo sapiens.” The analysis was performed with a “medium confidence” minimum required interaction score (>0.4). The resulting data were then imported into a network biology visualization analysis, where Cytoscape software version 3.10.0 was employed to calculate the parameters of each node in the network diagram, representing molecular interactions. To identify the core targets, the CytoHubba_MCC algorithm was utilized for network analysis. Additionally, MCODE plug-in analysis highlighted eight key genes within the most significant module.

### 2.8. Functional Annotation and Pathway Enrichment Analysis of Target Proteins

To explore the biological functions of the potential targets of Nd_2_O_3_ in pulmonary fibrosis, data from the Metascape and STRING databases were integrated. A comprehensive Gene Ontology (GO) analysis, which encompassed biological processes, cellular components, and molecular functions, was performed to provide valuable insights into the functional roles of these targets. Additionally, KEGG pathway enrichment analysis was conducted to identify key pathways associated with Nd_2_O_3_-mediated pulmonary fibrosis, focusing on both therapeutic and toxic pathways to gain a deeper understanding of the underlying mechanisms. These analyses aim to enhance our understanding of the molecular basis of Nd_2_O_3_-induced lung injury.

### 2.9. Molecular Docking

To provide structural support for selected target proteins identified through network toxicology analysis, molecular docking was performed using representative reference inhibitors with known binding preferences. The three-dimensional structures of the reference ligands were obtained from PubChem, and the structures of the target proteins were retrieved from the AlphaFold Protein Structure Database. Protein structures were prepared in PyMOL version 2.5.5 by removing water molecules and original ligands, followed by hydrogen addition and charge assignment. Docking analysis was then performed using CBDock Vina. The docking poses and intermolecular interactions were visualized using Discovery Studio and PyMOL.

### 2.10. Metabolomics

Metabolomic profiling was performed by Hangzhou Lianchuan Biotechnology Co., Ltd. (Hangzhou, China). Approximately 50 mg (±5 mg) of lung tissue from each mouse in the control group and the 1000 mg/m^3^ Nd_2_O_3_ exposure group (*n* = 3 per group) was extracted with 500 μL of pre-chilled 80% methanol and homogenized with stainless-steel beads. Homogenates were incubated at −20 °C for 30 min to precipitate proteins and centrifuged at 20,000× *g* for 10 min, followed by an additional 5 min centrifugation. The supernatants were transferred to fresh vials for UPLC–HRMS analysis, and pooled quality control (QC) samples prepared from all samples were used to monitor system stability. LC–MS raw data were converted to mzXML format and processed in R using XCMS for feature detection and alignment, with CAMERA and metaX used for isotope/adduct annotation and downstream preprocessing. The workflow included peak picking, grouping, retention time correction, and re-grouping. Metabolic features were defined by unique retention time–*m*/*z* pairs, generating a features × samples matrix for subsequent statistical analysis. Differential metabolites were screened based on FC > 1.5 or <0.67, OPLS-DA VIP > 1, and adjusted *p* < 0.05. Multivariate analyses, including PCA, OPLS-DA, hierarchical clustering, and pathway enrichment analysis, were performed to visualize global metabolic differences and interpret their biological significance.

### 2.11. RNA Extraction and RT-qPCR

Total RNA was extracted from mouse lung tissues using TRIzol reagent, and RNA yield and purity were determined by spectrophotometry (A260/A280). Complementary DNA (cDNA) was synthesized with a reverse transcription kit from Vazyme Biotech (Nanjing, China). Quantitative real-time PCR (qRT-PCR) was carried out using GoTaq^®^ qPCR Master Mix (Promega, Madison, WI, USA), and fluorescence signals were collected on a QuantStudio™ 5 Real-Time PCR System (Thermo Fisher Scientific, Waltham, MA, USA). GAPDH was used as the endogenous control for normalization, with technical replicates performed for each sample. Relative transcript abundance was calculated using the 2^−ΔΔCt^ method to evaluate differences in target gene expression between groups. The primer sequences used in this study are listed in Table 1.

### 2.12. Statistical Analysis

All statistical analyses were performed using SPSS 24.0 software. Data are presented as the mean ± standard deviation (SD). Each experiment was performed with at least three independent samples or biological replicates unless otherwise stated. The normality of data distribution was assessed using the Shapiro–Wilk test, and homogeneity of variance was evaluated before comparisons among groups. For comparisons between two groups, Student’s *t*-test was used. For comparisons among multiple groups, one-way analysis of variance (ANOVA) was performed, followed by Bonferroni’s post hoc test for pairwise comparisons. If the data did not meet the assumptions for parametric analysis, a non-parametric test was used as appropriate. A value of *p* < 0.05 was considered statistically significant. Western blot bands were quantified using ImageJ software and normalized to GAPDH. GraphPad Prism 10.0 was used for data visualization.

## 3. Results

### 3.1. Nd_2_O_3_ Exposure Induced Histopathological Injury and Fibrosis-Related Changes in Lung Tissue

**Figure 1:**
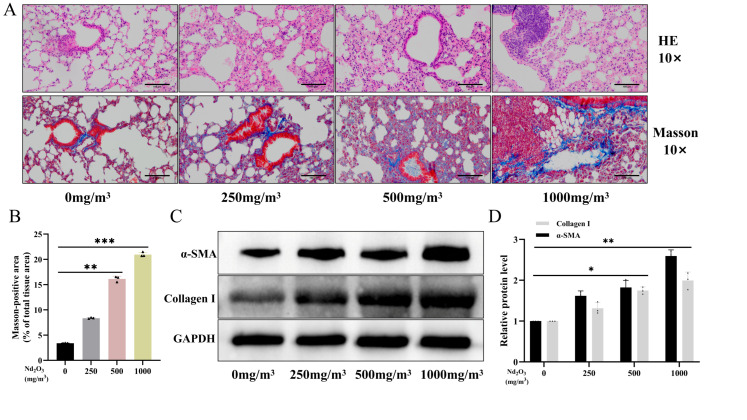
Nd_2_O_3_ exposure induced lung histopathological injury and fibrosis-related changes in mice. (**A**) Representative histological images of lung tissues stained with hematoxylin and eosin (HE) and Masson’s trichrome in the control and Nd_2_O_3_-exposed groups (0, 250, 500, and 1000 mg/m^3^). HE staining showed progressive lung injury characterized by alveolar septal thickening, focal mononuclear cell accumulation, lymphoid follicle-like aggregates, and disruption of pulmonary architecture. Masson’s trichrome staining showed increased collagen fiber deposition in lung tissues following Nd_2_O_3_ exposure. Scale bar = 100 μm. (**B**) Semi-quantitative histomorphometric analysis of Masson’s trichrome-positive collagen areas in lung parenchymal regions, excluding large bronchi and blood vessels. Triangles indicate data points. (**C**) Representative Western blot bands of α-SMA, Collagen I, and GAPDH in lung tissues from different exposure groups. (**D**) Relative protein expression levels of α-SMA and Collagen I normalized to GAPDH. Data are presented as mean ± SD. * *p* < 0.05, ** *p* < 0.01, *** *p* < 0.001 versus the control group.

Histopathological examination revealed that Nd_2_O_3_ exposure induced progressive lung injury and fibrosis-related changes in mice. H&E staining showed that the control group maintained normal lung architecture, with intact alveolar structures and no obvious histopathological abnormalities. In contrast, Nd_2_O_3_-exposed groups exhibited dose-related histopathological alterations, including alveolar septal thickening, focal mononuclear cell accumulation, focal lymphoid follicle-like aggregates, and disruption of pulmonary architecture, with the most severe lesions observed in the 1000 mg/m^3^ group (Figure 1A). Based on H&E morphology, these focal cellular aggregates appeared predominantly mononuclear and lymphocyte-like. With increasing exposure concentration, mononuclear cell accumulation became more evident, and focal lymphoid follicle-like aggregates were observed in the 1000 mg/m^3^ group. Trichrome staining revealed a concentration-dependent increase in collagen deposition following Nd_2_O_3_ exposure, particularly within the pulmonary interstitium and the peribronchial and perivascular regions, with the highest level of deposition observed in the 1000 mg/m^3^ group (Figure 1A). Consistent with the histological observations, semi-quantitative histomorphometric analysis using ImageJ/Fiji revealed a significant concentration-dependent increase in the percentage of collagen-positive area within lung parenchymal regions, excluding large bronchi and blood vessels, following Nd_2_O_3_ exposure (Figure 1B). Western blot analysis further showed that the protein expression levels of α-SMA and Collagen I were elevated in the lungs of Nd_2_O_3_-exposed mice compared with those in the control group, with more marked increases at higher exposure concentrations (Figure 1C,D). These results indicate that Nd_2_O_3_ exposure promotes lung injury accompanied by focal mononuclear cell accumulation, lymphoid follicle-like aggregates, enhanced collagen deposition, and fibrosis-related molecular alterations.

### 3.2. Identification of Targets of Nd_2_O_3_ Affecting Pulmonary Fibrosis

**Figure 2:**
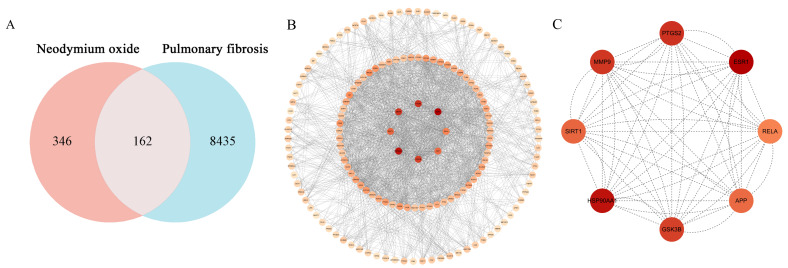
Network toxicology analysis and potential target identification for Nd_2_O_3_-induced pulmonary fibrosis. (**A**) Venn diagram of Nd_2_O_3_-related and pulmonary fibrosis-related targets, showing 162 overlapping targets, 346 Nd_2_O_3_-specific targets, and 8435 pulmonary fibrosis-specific targets. (**B**) Protein–protein interaction (PPI) network of the 162 overlapping targets. Nodes represent protein targets and edges represent protein–protein interactions; darker and more central nodes indicate higher connectivity. (**C**) Core target subnetwork showing the top-ranked hub genes identified from the PPI network analysis, including *ESR1*, PTGS2, *HSP90AA1*, *MMP9*, *GSK3B*, *SIRT1*, *APP*, and *RELA*.

A total of 508 putative Nd_2_O_3_-related targets were obtained from the ChEMBL, TargetNet, and SuperPred databases. Pulmonary fibrosis-related targets were collected from GeneCards, DrugBank, TTD, DisGeNET, and OMIM, resulting in 8597 disease-associated targets. After integration and removal of redundant targets, 162 overlapping targets were identified as potential targets involved in Nd_2_O_3_-induced pulmonary fibrosis. Figure 2A shows the overlapping targets between Nd_2_O_3_ and pulmonary fibrosis. A protein–protein interaction (PPI) network was then constructed using the STRING database, which contained 159 nodes and 959 edges. Cytoscape was used to analyze the topological characteristics of the network and to construct the PPI network (Figure 2B). Based on degree values, the top four core targets were *ESR1*, *PTGS2*, *HSP90AA1*, and *MMP9*. Because the initial target screening and PPI analysis were based mainly on human-derived databases, the network-derived targets were considered putative candidates and were further aligned with the mouse experimental model through ortholog mapping. To improve species consistency with the mouse experimental model, the four topological core targets identified from the human-based PPI network were mapped to their corresponding Mus musculus orthologs using the Mouse Genome Informatics database and the HGNC Comparison of Orthology Predictions database. *ESR1*, *PTGS2*, *HSP90AA1*, and *MMP9* were confirmed to correspond to the mouse orthologs *Esr1*, *Ptgs2*, *Hsp90aa1*, and *Mmp9*, respectively. Previous studies have reported that these targets are associated with pulmonary fibrosis-related pathological processes. Previous studies have implicated these targets in pulmonary fibrosis-related pathological processes, although their biological roles may differ. *ESR1* has been reported to exert protective effects in the lung, and its expression is reduced in end-stage idiopathic pulmonary fibrosis (IPF) tissues and suppressed by transforming growth factor-β1 (TGF-β1) in bronchial epithelial cells [23]. Similarly, *PTGS2* (COX-2) is generally considered an anti-fibrotic mediator, and reduced PTGS2 inducibility in IPF fibroblasts may contribute to decreased prostaglandin E2 production and enhanced fibrogenic responses [24]. In contrast, *HSP90AA1* encodes HSP90α, and HSP90-related signaling has been associated with fibroblast activation, extracellular matrix deposition, and pulmonary fibrosis progression [25]. *MMP9* is frequently upregulated in IPF lungs and participates in extracellular matrix remodeling and TGF-β-related fibrotic responses [26]. Taken together, *ESR1*, *PTGS2*, *HSP90AA1*, and *MMP9* were prioritized as network-derived hub targets potentially associated with Nd_2_O_3_-induced fibrosis-related lung responses. However, because PPI network analysis reflects topological importance rather than exposure-induced expression changes, these targets were interpreted as putative candidates and were further evaluated together with downstream experimental validation.

### 3.3. Target Analysis and Pathway Enrichment Analysis

**Figure 3:**
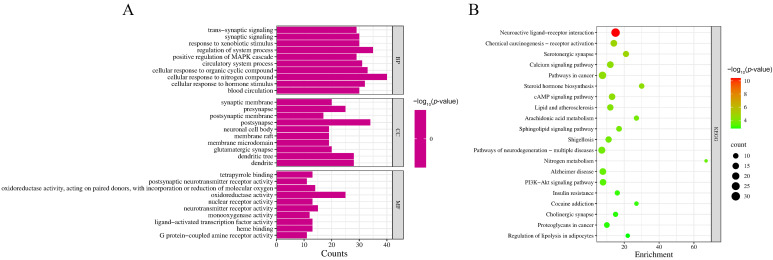
GO and KEGG enrichment analyses of the overlapping targets. (**A**) GO enrichment analysis of the overlapping targets, showing the top enriched terms in the biological process (BP), cellular component (CC), and molecular function (MF) categories. (**B**) KEGG pathway enrichment analysis of the overlapping targets, showing the top enriched pathways. Dot size represents target count.

We use the Metascape database to conduct further GO analysis on the 162 potential targets in Homo sapiens, resulting in 1603 statistically significant GO terms. These terms were classified under 1294 BP, 109 CC, and 200 MF. GO terms were ranked based on their *p* value to identify most important terms and in each category (BP, CC and MF), top 10 terms were selected for further analysis. These selected terms were then plotted as an enrichment analysis diagram in Figure 3A, which provides a clear visualization of the analysis results. In addition, KEGG pathway enrichment analysis using the Metascape database identified a total of 144 enriched signaling pathways, and the results are presented as a bubble chart (Figure 3B). Figure 3B shows the top 20 KEGG pathways ranked by *p*-value and provides more detailed information on the pathways involved in this study. As is seen from the GO and KEGG analysis of potential targets, a significant term appeared in the GO, which was the positive regulation of the MAPK cascade. KEGG pathway enrichment analysis highlighted several enriched pathways, including neuroactive ligand–receptor interaction, chemical carcinogenesis–receptor activation, and the cAMP signaling pathway, some of which may be relevant to pulmonary fibrosis.

### 3.4. Molecular Docking Validation of Selected Target Proteins Using Reference Inhibitors

**Figure 4:**
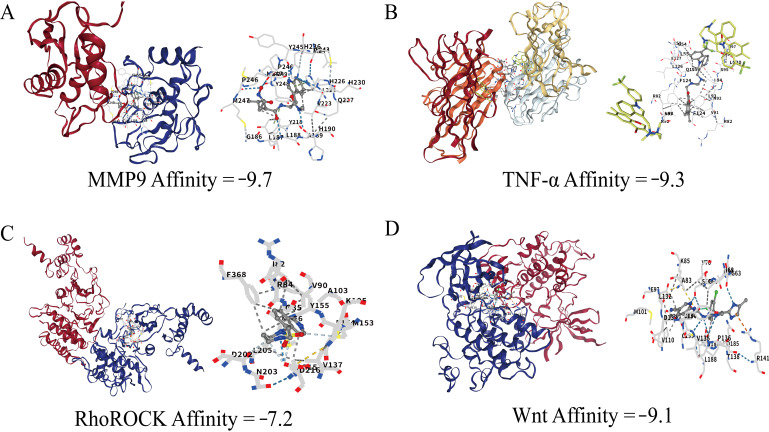
Molecular docking results of potential targets. (**A**–**D**) Docking models of the reference inhibitors with *MMP9*, *TNF-α*, *ROCK1*, and *GSK3β*, respectively. The left panels show the overall binding modes, and the right panels show the interactions between the ligand and key amino acid residues. Binding affinity values (kcal/mol) are indicated for each target.

In the molecular docking analysis, TNF-α, *MMP9*, ROCK1, and GSK3β were used as representative targets because they were present in the overlapping target set and previous studies have demonstrated their important roles in pulmonary fibrosis-associated processes or pathways, including inflammatory regulation, extracellular matrix remodeling, MAPK-related signaling, and injury–repair responses. Molecular docking simulations indicated stable and chemically plausible interactions between key targets and their respective classical inhibitors. SPD304 was accommodated at the TNF-α trimer interface with a predicted binding energy of −9.3 kcal/mol, primarily stabilized by hydrophobic interactions and π–π contacts. GM6001 exhibited strong affinity toward the catalytic domain of *MMP9* (−9.7 kcal/mol), coordinating the active-site Zn^2+^ and forming hydrogen-bond interactions with key residues such as His401 and Glu402. Fasudil docked into the ATP-binding pocket of ROCK1 with a binding energy of −7.2 kcal/mol, generating hinge-region hydrogen bonds together with hydrophobic contacts that support a canonical kinase-pocket binding mode. Similarly, CHIR99021 occupied the ATP site of GSK3β (−9.1 kcal/mol) and established dual hinge hydrogen bonds with Val135 and Asp133 (Figure 4). Collectively, the binding poses and interaction patterns were consistent with the expected pharmacophoric features of these reference inhibitors, supporting the suitability of the docking protocol and highlighting the druggability of TNF-α, *MMP9*, ROCK1, and GSK3β. When integrated with the network toxicology-based prioritization, these proteins were therefore retained as candidate targets potentially implicated in Nd_2_O_3_-induced pulmonary fibrosis and warrant further mechanistic validation in downstream experiments.

### 3.5. Metabolic Characteristics of Mouse Lungs After Nd_2_O_3_ Exposure

**Figure 5:**
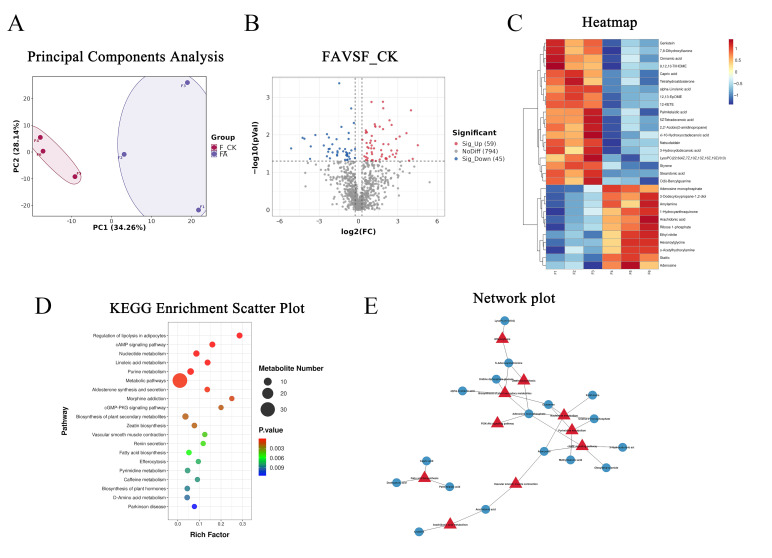
Metabolomic alterations in mouse lungs following Nd_2_O_3_ exposure. (**A**) Principal component analysis (PCA) showing the overall metabolic separation between the control and Nd_2_O_3_ groups. (**B**) Volcano plot of differential metabolites between the control and Nd_2_O_3_ groups. (**C**) Heatmap showing the relative abundance patterns of differential metabolites in the control and Nd_2_O_3_ groups. (**D**) KEGG enrichment analysis showing the major metabolic pathways associated with the differential metabolites. (**E**) Metabolite–pathway association network illustrating the relationships between altered metabolites and enriched pathways.

PCA revealed that Nd_2_O_3_ exposure induced significant metabolic changes in the lungs of mice (Figure 5A). A total of 104 differentially expressed metabolites were identified through differential metabolite analysis, among which 59 metabolites were up-regulated and 45 metabolites were down-regulated after Nd_2_O_3_ exposure (Figure 5B). Meanwhile, the results of cluster analysis showed the difference between the two groups more clearly, and the metabolites that were highly expressed in the control group were reversed in the Nd_2_O_3_ group (Figure 5C). KEGG enrichment analysis revealed that these differential metabolites were primarily involved in key biological processes, including regulation of lipolysis in adipocytes, cAMP signaling pathway, nucleotide metabolism, renin secretion, and fatty acid biosynthesis (Figure 5D). A metabolite–pathway association network was generated by mapping differential metabolites to KEGG pathways (dots denote metabolites, triangles denote enriched pathways, and edges indicate pathway membership) (Figure 5E). Nucleotide/pyrimidine metabolism constituted the central module and was linked to the PI3K–Akt and cAMP signaling pathways through metabolites associated with adenosine, such as adenosine, AMP, and UMP, suggesting a strong connection between metabolic disturbances and the regulation of signaling pathways. In parallel, lipid-related pathways, including fatty acid biosynthesis and arachidonic acid metabolism, formed relatively independent clusters, suggesting concomitant remodeling of lipid metabolism. These findings indicated that Nd_2_O_3_ exposure disrupted the basic metabolic pathways associated with Regulation of Lipolysis, Fatty Acid Biosynthesis, Nucleotide Metabolism, and Amino Acid Metabolism, which may result in pulmonary dysfunction and fibrosis.

### 3.6. Integrative Analysis of Network Toxicology and Metabolomics

**Figure 6:**
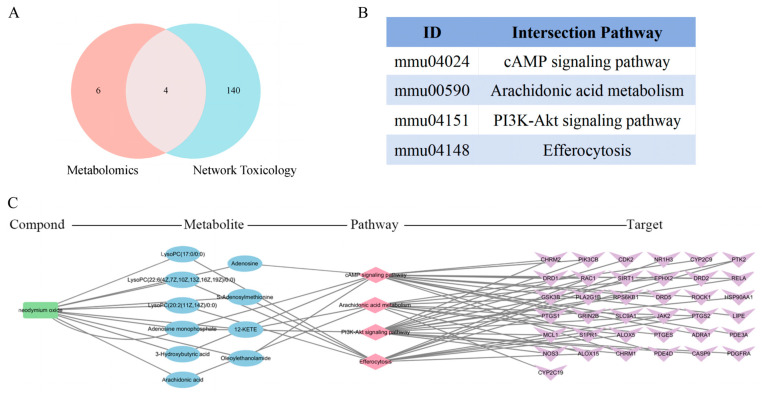
Integrated analysis of network toxicology and metabolomics. (**A**) Venn diagram showing the overlap between pathways identified by metabolomics and network toxicology analyses, with 4 shared pathways. (**B**) The four overlapping pathways identified by the integrated analysis: cAMP signaling pathway, arachidonic acid metabolism, PI3K–Akt signaling pathway, and efferocytosis. (**C**) Compound–metabolite–pathway–target network constructed from the integrated analysis. The network illustrates the relationships among Nd_2_O_3_, differential metabolites, overlapping pathways, and potential target genes.

Four key metabolic pathways in metabolomics were mapped onto network toxicology pathways for analysis (Figure 6A). These 10 metabolomic pathways were identified by pathway enrichment analysis of the differential metabolites and are shown in the metabolite–pathway association network in Figure 5E. The results demonstrated that the cAMP signaling pathway, arachidonic acid metabolism, PI3K-Akt signaling pathway, and efferocytosis were enriched pathways in the intersection of the two analyses, suggesting that the study subjects may primarily influence the metabolism of inflammatory mediators, cell survival/stress signal transduction, and phagocytic clearance processes related to inflammation resolution (Figure 6B). To better characterize system-level links among compound perturbation, metabolic changes, pathway modulation, and target engagement, we built a multilayer compound–metabolite–signaling pathway–target network (Figure 6C). The resulting network showed that differential metabolites and predicted targets clustered in a pathway-driven manner and were mainly assigned to four shared pathways: the cAMP signaling pathway, arachidonic acid metabolism, the PI3K–Akt signaling pathway, and efferocytosis. Together, these pathways formed the principal connections bridging the compound with metabolic alterations and target-associated interactions, prioritizing them for downstream identification of key metabolites/targets and targeted mechanistic validation.

### 3.7. qPCR Analysis of Key Target Genes

**Figure 7:**
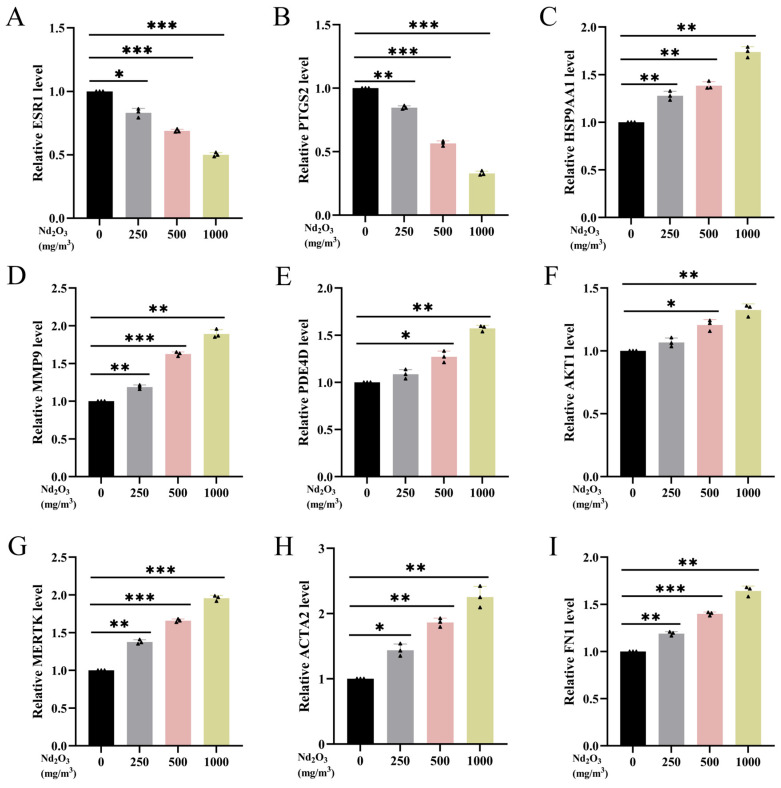
Relative mRNA expression levels of ESR1, PTGS2, HSP90AA1, MMP9, PDE4D, AKT1, MERTK, ACTA2, and FN1 in lung tissues from mice exposed to Nd_2_O_3_. ESR1 and PTGS2 transcripts tended to decrease, whereas HSP90AA1, MMP9, PDE4D, AKT1, MERTK, ACTA2, and FN1 tended to increase across the 250, 500, and 1000 mg/m^3^ exposure groups (**A**–**I**). Data are presented as mean ± SD. * *p* < 0.05, ** *p* < 0.01, *** *p* < 0.001 vs. the 0 mg/m^3^ group. Triangles indicate individual data points.

Based on integrated network toxicology and metabolomics analyses, key targets and the associated pathways were identified, and pathway-related candidate genes were selected for validation by RT–qPCR. The candidate set included hub targets identified by network toxicology, namely *ESR1*, *PTGS2*, *HSP90AA1*, and *MMP9*. In parallel, the integrated analysis highlighted the concurrent enrichment of the cAMP signaling pathway, arachidonic acid metabolism, the PI3K–Akt signaling pathway, and efferocytosis. To obtain pathway-level transcriptional readouts with greater specificity, one representative node gene was chosen for each pathway: *PDE4D* (cAMP), *PTGS2* (arachidonic acid metabolism), *AKT1* (PI3K–Akt), and *MERTK* (efferocytosis). In addition, to probe potential fibrosis-associated molecular responses, we quantified the transcript levels of the fibrosis markers *ACTA2* and *FN1*, providing phenotype-relevant molecular evidence. Finally, RT-qPCR was performed on lung tissue samples from the control, 250, 500, and 1000 mg/m^3^ exposure groups to verify exposure-associated changes in mRNA expression for these targets. The RT-qPCR data showed that, compared with the control group, *ESR1* and *PTGS2* mRNA expression levels were decreased in Nd_2_O_3_-exposed mice, whereas *HSP90AA1*, *MMP9*, *PDE4D*, *AKT1*, *MERTK*, *ACTA2*, and *FN1* were increased, particularly in the higher exposure groups (Figure 7A–I). These transcriptional alterations were generally consistent with the network toxicology and metabolomics analyses and may be associated with changes in PI3K–Akt signaling, cAMP-related regulation, efferocytosis-associated responses, and fibrosis-related biological processes following Nd_2_O_3_ exposure. However, the functional significance of these gene expression changes and their direct involvement in the development of pulmonary fibrosis require further experimental validation.

## 4. Discussion

Rare earth elements are strategic resources widely utilized across various industries, including manufacturing, agriculture, military, environmental, and medical sectors. They also have significant applications in biomedical imaging and as antitumor agents [27]. Nd_2_O_3_, a commonly used rare-earth material, is integral to the production of glass, capacitors, and magnets. However, exposure to Nd_2_O_3_ has been linked to adverse health effects, including pulmonary fibrosis [15,16,28]. Studies of occupational exposure have revealed fibrotic-like changes in the lung tissue of workers with prolonged contact with Nd_2_O_3_. Furthermore, clinical investigations have shown that chest CT scans of Nd_2_O_3_-exposed workers often display significant ground-glass opacities, primarily manifested as small nodular shadows, suggesting that exposure to Nd_2_O_3_ may induce pneumoconiosis. However, a systematic investigation is warranted to elucidate the cytotoxicity of Nd_2_O_3_ and its underlying molecular mechanisms in the pathogenesis of pulmonary fibrosis. In the present study, HE staining demonstrated obvious pathological alterations in the lungs of Nd_2_O_3_-exposed mice, Masson staining revealed increased collagen fiber deposition, and the upregulation of fibrosis-related proteins further supported the presence of fibrosis-related changes in lung tissue. It should be noted that the exposure concentrations used in the present study, 250–1000 mg/m^3^, are higher than typical occupational exposure levels. Therefore, this exposure paradigm is best interpreted as a high-dose hazard-identification model rather than a direct reproduction of routine workplace exposure conditions. Nevertheless, the exposure-related histopathological changes, collagen deposition, and fibrosis-related protein expression observed across the tested concentrations support the biological relevance of this model. Future studies incorporating lower, occupationally relevant exposure concentrations are warranted to further validate the dose–response relationship and improve the extrapolation of these findings to real-world occupational exposure scenarios.

Network toxicology leverages existing databases for high-throughput screening and prediction and has strong hypothesis-generating capability, but its results are often limited to associations inferred from in silico simulations [29,30]. Metabolomics directly reflects the overall changes in endogenous metabolites in an organism under specific stimuli, serving as a powerful tool for revealing terminal phenotypic and functional alterations [31]. By integrating the two approaches, this study systematically investigated the molecular mechanisms underlying Nd_2_O_3_-induced pulmonary fibrosis. Initially, network toxicology analysis identified candidate toxicity-associated genes and enriched signaling pathways, including the cAMP and PI3K–Akt pathways, potentially implicated in Nd_2_O_3_-mediated pulmonary injury. Subsequently, untargeted metabolomics profiling was performed to independently validate and functionally contextualize these bioinformatically derived pathways. Notably, arachidonic acid metabolism was significantly perturbed, and additional dysregulated metabolites linked to lipid inflammation and intercellular signal transduction were identified. Finally, RT-qPCR validation confirmed differential expression of key pathway-related genes: *ESR1* and *PTGS2* were significantly downregulated, whereas *HSP90AA1*, *MMP9*, *PDE4D*, *AKT1*, and *MERTK* were significantly upregulated. These experimental findings corroborate the bioinformatic predictions and metabolomic observations, collectively supporting the biological plausibility of the proposed mechanistic framework.

Our analysis identified *ESR1*, *PTGS2*, *HSP90AA1*, and *MMP9* as key hub genes. Their expression changes in lung tissues following Nd_2_O_3_ exposure, downregulation of *ESR1* and *PTGS2,* and upregulation of *HSP90AA1* and *MMP9* were experimentally confirmed, indicating that these genes likely play important roles in Nd_2_O_3_-induced lung injury. By integrating network toxicology and metabolomics analyses, Nd_2_O_3_ exposure may induce fibrosis-like molecular responses in lung tissue by disrupting pathways related to cAMP signaling, arachidonic acid metabolism, PI3K–Akt signaling, and phagocytic clearance. Moreover, our findings suggest that Nd_2_O_3_ exposure may promote fibrosis-related lung responses through the PI3K–Akt pathway, which is consistent with our previous findings [32]. This multi-omics integration strategy not only elucidates the mechanistic basis of Nd_2_O_3_-induced pulmonary toxicity but also provides a robust foundation for the identification of early biomarkers and the discovery of intervention targets.

*ESR1* is widely expressed in lung tissue and exerts anti-inflammatory, antioxidant, and anti-fibrotic effects [33]. Studies have shown that estrogen signaling can alleviate pulmonary fibrosis by inhibiting TGF-β pathway and reducing collagen deposition [34]. This study found that the expression of *ESR1* mRNA in lung tissue was significantly downregulated after exposure to Nd2O3, which may indicate that an endogenous anti-fibrotic protective mechanism was weakened or shut down. Downregulation of *ESR1* may weaken its inhibitory capacity toward downstream pro-fibrotic signaling, such as TGF-β, thereby creating a microenvironment conducive to fibrotic progression. This provides new molecular clues for understanding why susceptibility to pneumoconiosis differs among certain occupationally exposed populations, potentially influenced by hormonal status. Furthermore, decreased *ESR1* expression may form a positive feedback loop with dysregulated arachidonic acid metabolism: impaired regulation further reduces PGE_2_ synthesis, whereas PGE_2_ can inhibit fibroblast activation by activating the cAMP–PKA pathway. This dual inhibition—both weakening estrogen receptor-mediated anti-fibrotic signaling and blocking cAMP-dependent negative regulation of fibrogenesis—may markedly amplify Nd_2_O_3_-induced lung tissue remodeling.

*PTGS2* is the key rate-limiting enzyme that catalyzes the conversion of arachidonic acid into PGE2. Our network analysis predicted the involvement of the arachidonic acid metabolism pathway, and the metabolomics data were consistent with perturbation of this pathway; meanwhile, RT-qPCR analysis showed that *PTGS2* gene expression was downregulated. Some studies have indicated that during the advanced stages of persistent inflammation or fibrosis, *PTGS2* expression is downregulated, resulting in an altered prostaglandin profile. Specifically, pro-fibrotic prostaglandins—such as PGF_2_α—are relatively elevated, whereas the anti-fibrotic prostaglandin PGE_2_ is significantly reduced [35]. Therefore, Nd_2_O_3_exposure may disrupt the balance of the arachidonic acid metabolic network, driving metabolic flux toward lipid mediators that promote inflammation and fibrosis rather than anti-inflammatory/anti-fibrotic mediators. Downregulation of *PTGS2* may serve as an important marker of this dysregulated state.

The protein encoded by *HSP90AA1* is known as heat shock protein 90α (HSP90α) and plays a central role in the cellular stress response, signal transduction, and protein stability. In a fibrotic milieu, HSP90α acts as a central mediator by stabilizing key proteins across multiple pro-fibrotic signaling pathways, such as TGF-β, PI3K–Akt, thereby promoting the activation of fibroblasts, the differentiation of myofibroblasts, and ECM deposition [36,37]. Upregulation of *HSP90AA1* expression in lung tissue following Nd_2_O_3_ exposure suggests the induction of a robust cellular stress response and may exacerbate injury by stabilizing pro-fibrotic signaling networks. Matrix metalloproteinase-9 (*MMP9*) can degrade ECM basement membrane components—including type IV collagen and plays dual roles in inflammatory cell recruitment, epithelial–mesenchymal transition (EMT), and tissue remodeling [38,39,40]. Early excessive activation of *MMP9* disrupts alveolar architecture and promotes inflammatory cell infiltration; at later stages, an imbalance between *MMP9* and tissue inhibitors of metalloproteinases (TIMPs) leads to aberrant ECM deposition. In the present study, network toxicology analysis together with RT–qPCR validation supported *MMP9* as a candidate target associated with Nd_2_O_3_-induced fibrosis-related lung responses, while docking analysis using reference inhibitors provided complementary structural support for selected target proteins. The upregulation of *MMP9* was consistent with increases in the fibrotic markers *ACTA2* (α-SMA) and fibronectin 1 (*FN1*), strongly suggesting that Nd_2_O_3_ exposure initiates an active ECM remodeling process, a central pathological feature of pulmonary fibrosis.

Both network toxicology and integrated bioinformatic analyses indicate that the cAMP signaling pathway and the PI3K–Akt signaling pathway are critically involved in the biological effects of Nd_2_O_3_. The cAMP serves as a critical intracellular second messenger. Diminished cAMP levels in pulmonary fibroblasts have been shown to enhance cellular proliferation and promote differentiation into myofibroblasts [41]. We verified the upregulation of *PDE4D* (phosphodiesterase 4D, a key enzyme responsible for cAMP degradation), which provides a direct mechanistic explanation for the attenuation of cAMP signaling. The PI3K–Akt pathway is a canonical pro-survival and pro-proliferative signaling cascade that is aberrantly activated in pulmonary fibrosis, thereby promoting fibroblast proliferation and activation [42]. Our data show that *AKT1* expression is upregulated. Interestingly, these two pathways engage in reciprocal crosstalk: the PI3K–Akt pathway can suppress cAMP production or signaling outputs, whereas cAMP signaling can negatively regulate Akt activity via protein kinase A (PKA) [43,44]. Exposure to Nd_2_O_3_ may concurrently suppress cAMP signaling through upregulation of *PDE4D*—and activate the PI3K–Akt signaling pathway, as evidenced by increased *AKT1* expression. This “dual hit” may synergistically disrupt signaling homeostasis, thereby strongly driving fibroblast activation and proliferation. This Nd_2_O_3_-triggered HSP90α–*PDE4D*–*AKT1* cooperative triad constitutes a highly integrated molecular hub in the progression of pulmonary fibrosis. These findings not only delineate the molecular cascade underlying Nd_2_O_3_-induced pulmonary fibrosis but also underscore the pivotal role of *HSP90AA1* as an upstream signaling hub.

The enrichment of the phagocytic clearance pathway is a noteworthy finding. We verified the upregulation of Mertk mRNA, which encodes *MERTK*, a receptor involved in the recognition and efferocytic clearance of apoptotic cells [45]. In the alveolar space, timely clearance of apoptotic alveolar epithelial and inflammatory cells is critical for inflammation resolution and for preventing persistent inflammation driven by secondary necrosis [45]. Activation of *MERTK*-mediated signaling is generally considered to facilitate apoptotic cell recognition and clearance, thereby contributing to inflammation resolution [46]. In the context of Nd_2_O_3_-induced lung injury, increased Mertk expression may therefore reflect activation of an efferocytosis-related response or a compensatory response to increased apoptotic cell burden [47,48]. Thus, the proposed link between increased Mertk expression and dysregulated injury–repair signaling should be considered hypothesis-generating and requires further functional validation. Further studies using phagocytosis assays, Gas6/ProS measurements, or *MERTK* inhibition experiments are needed to determine whether increased Mertk expression reflects effective efferocytosis or is associated with dysregulated injury–repair responses in the lung.

Metabolomic analyses showed that the differential metabolites were significantly enriched in pathways related to lipid metabolism and inflammation. Perturbations in arachidonic acid metabolism have been described above. In addition, changes in other lipid mediators, such as sphingolipids and phospholipids, may affect the integrity of cell membranes, signal transduction, and the activation of inflammasomes; these lipids can themselves act as signaling molecules that exacerbate inflammatory and fibrotic responses [49,50]. Collectively, the metabolomic data suggest that Nd_2_O_3_ exposure was associated with metabolic alterations in lung tissue that may be related to inflammatory and fibrotic responses. Because lipid- and inflammation-related metabolic pathways are broadly involved in tissue injury and remodeling, their interpretation in this study should be considered in the context of Nd_2_O_3_-exposed lung tissue and the accompanying pathological changes. These metabolomic findings may reflect metabolic disturbances associated with pulmonary inflammation and fibrotic remodeling after Nd_2_O_3_ exposure.

This study also has several limitations. First, the network toxicology predictions were based on currently available databases, which may result in false-positive or false-negative target identification. Second, although the present study integrated network toxicology, metabolomics, and experimental validation to explore the potential mechanisms underlying Nd_2_O_3_-induced pulmonary fibrosis, validation of key molecular targets was primarily conducted at the transcriptional level. Additional protein-level analyses are needed to further confirm the biological roles of these targets. Third, the biodistribution and tissue burden of Nd_2_O_3_ nanoparticles were not evaluated in the current study. Future investigations incorporating toxicokinetic analyses, including particle retention, clearance, and systemic distribution, will contribute to a more comprehensive understanding of the biological effects of Nd_2_O_3_ nanoparticles.

## 5. Conclusions

This study employed an integrative strategy combining network toxicology and metabolomics to systematically characterize molecular alterations in the lung following Nd_2_O_3_ exposure and to generate mechanistic hypotheses related to pro-fibrotic responses. Through a multi-database mining workflow, *ESR1*, *PTGS2*, *HSP90AA1*, and *MMP9* were prioritized as hub genes. Pathway enrichment analyses consistently highlighted the cAMP signaling pathway, arachidonic acid metabolism, the PI3K–Akt signaling pathway, and phagocytosis-related clearance processes as key biological modules. Collectively, the mechanisms potentially involved in Nd_2_O_3_-induced fibrosis-related lung responses may include: suppression of cAMP signaling via *PDE4D* upregulation, which acts synergistically with activation of the PI3K–Akt pathway driven by *AKT1* upregulation to promote fibroblast activation; disruption of lipid metabolism, including arachidonic acid metabolism, thereby facilitating the generation of pro-fibrotic lipid mediators; induction of cellular stress (*HSP90AA1* upregulation) and protease imbalance (*MMP9* upregulation), leading to aberrant ECM remodeling; and potential impairment of alveolar clearance function (*MERTK* upregulation). These pathways are intertwined and together constitute a systems-biology landscape of Nd_2_O_3_-induced pulmonary toxicity. These findings provide a multi-omics, network-based framework for understanding the potential pulmonary toxicity of Nd_2_O_3_ and identify candidate targets for subsequent mechanistic investigations and intervention-oriented studies.

## Figures and Tables

**Table 1:** Primer Sequences.

Genes	Sequences
*ESR1*	F:TCTGCCAAGGAGACTCGCTACT
	R:GGTGCATTGGTTTGTAGCTGGAC
*PTGS2*	F:GCGACATACTCAAGCAGGAGCA
	R:AGTGGTAACCGCTCAGGTGTTG
*HSP90AA1*	F:GCTTTCAGAGCTGTTGCGGTAC
	R:AAAGGCGGAGTTAGCAACCTGG
*MMP9*	F:GCTGACTACGATAAGGACGGCA
	R:TAGTGGTGCAGGCAGAGTAGGA
*PDE4D*	F:CACAGACTTGGAGATTCTCGCG
	R:TCTAGGACCGAGGAGTCGTTGT
*AKT1*	F:GGACTACTTGCACTCCGAGAAG
	R:CATAGTGGCACCGTCCTTGATC
*MERTK*	F:ATCATCCTCGGCTGCTTCTGTG
	R:ACGACCAGTTGGGAATCCTCCT
*ACTA2*	F:TGCTGACAGAGGCACCACTGAA
	R:CAGTTGTACGTCCAGAGGCATAG
*FN1*	F:CCCTATCTCTGATACCGTTGTCC
	R:TGCCGCAACTACTGTGATTCGG
*GAPDH*	F:GAAAGCCTGCCGGTGACTAA
	R:AGGAAAAGCATCACCCGGAG

## Data Availability

The original data presented in this study are included in the article/Supplementary Materials; further inquiries can be directed to the corresponding author.

## Supplementary Materials

The following supporting information can be downloaded at: https://www.mdpi.com/article/10.3390/toxics14060513/s1. Supplementary Material S1: Completed ARRIVE guidelines 2.0 author checklist.
